# Current News

**Published:** 1900-04

**Authors:** 


### Current News.

### IMPORTANT NOTICE.

At the last meeting of the Dental Commissioners of Connecticut it was

*Voted,* That after January 1, 1900, every applicant for license, whether graduate or non-graduate, be required to pass a thorough examination, both theoretical and practical, the details of which shall be arranged later, and that all rules conflicting therewith be and are hereby repealed.

The recorder was requested to devise some plan whereby operations can be performed by applicants upon patients before the commissioners.

Full particulars as to the required examination will be given each candidate as soon as possible before the next examination, which will be held Monday, May 21, 1900, at the Capitol in Hartford. It must be distinctly understood by all applicants who receive a temporary permit after January 1, 1900, that the examination passed to obtain the same does not exempt them from passing the regular examination for license at the next meeting of the commission.

GEORGE L. PARMELE,
*Dental Commissioner and Recorder.*

HARTFORD, CONN., January 1, 1900.

---

### UNITED STATES CIRCUIT COURT, SOUTHERN DISTRICT OF NEW YORK.

### THE INTERNATIONAL TOOTH-CROWN COMPANY *vs.* JAMES ORR KYLE.

TOWNSEND, District Judge. Final hearing on bill and answer raising questions of validity and infringement of complainant's Patent No. 238,940, issued March 15, 1881, to James E. Low, its assignor, for an improvement in dentistry.

This patent has already been before this court in the suit of this complainant against Richmond, 30 Fed. 775, where the patent was sustained, and in its suit *vs.* Bennett, 77 Fed. 313, where the Circuit Court and Court of Appeals held that the patent was anticipated. The opinions in said suits show the character of the patented invention and discuss the issues involved. On the argument of this case the two defences presented were denial of infringement and anticipation. The claims alleged to be infringed are the following:

"1. The herein-described method of inserting and supporting artificial teeth, which consists in attaching said artificial teeth to continuous bands fitted and cemented to the adjoining and permanent teeth, whereby said artificial teeth are supported by said permanent teeth without dependence upon the gum beneath.

"2. An artificial tooth cut away at the back, so as not to present any contact with the gum, except along its front lower edge, and supported by rigid attachment to one or more adjoining permanent teeth, substantially as and for the purpose set forth."

The admission of defendant as to infringement is as follows:

"It is admitted by defendant that within the period of two years last past, and prior to the commencement of this suit, in the City of New York, N. Y., in the regular course of his professional work, he performed an operation in dentistry, in all respects similar to that illustrated by the model marked 'Model of Infringing Operation,' of which the following is a description:

"Inserting and supporting artificial teeth in the mouth of a patient by attaching said artificial teeth to continuous bands fitted and cemented to the adjoining permanent teeth, whereby said artificial teeth were supported by said permanent teeth without dependence upon the gum beneath; each of these artificial teeth in this operation was cut away at the back, so as not to present any contact with the gum, except along its front lower edge."

The exhibit which illustrates said operation clearly shows infringement, especially as such described method and completed structure come within the specification and claim of the patent in suit as construed by Judges Wallace and Shipman in the Richmond case. The following extract from said opinion sufficiently establishes this point:

"By the method of the patent a plate is dispensed with when some natural teeth remain, and, instead of the artificial teeth being loosely clasped to the adjacent natural teeth, they are attached with strength and permanency, and are not forced into contact with the gum during the strain of mastication. . . . When the artificial teeth employed have their surface adjacent to the gum cut away at the back, and only descend to contact with the gum along the front edge, another advantage results; because the small area covered by the bases of the teeth precludes such an accumulation of food or other foreign matter between the gum and the denture as cannot be readily removed.

"The second claim includes with the elements of the first claim, the features of a tooth cut away at the back. Thus construed defendants infringe both claims of the patent."

The first contention in support of the defence of anticipation is, that there were two applications for two distinct inventions, and that the latter invention, not conceived until after May, 1880, was anticipated.

Counsel for defendant says,—

"Low's first application was simply a wall over a space, having and resting upon the jaw for a foundation. His second application upon which patent in suit was granted, was a bridge thrown across a space, not only held in place, but supported by the abutment, or abutments, without gaining any support otherwise. An entirely different principle."

But this precise question was before the judges in the Richmond case, and was exhaustively discussed and finally disposed of as appears from the following citation:

"The defence is relied on that the invention had been in public use for more than two years before the application for the patent. The proofs show that operations were performed by Low during the latter part of the year 1877, in which he inserted the dentures of the patent in the mouths of patients. As the application upon which the patent was granted was not filed until December 20, 1880, the defence would be established were it not for the fact that Low had made an application, which was filed in the Patent Office January 6, 1879, which had never been abandoned, for substantially the same invention. That application contained some matters foreign to the subject of the second application, but, so far as it related to the inventions covered by the claim of the patent, it did not differ from the second application, except in a single particular. The specification of the patent states that non-contact of the artificial tooth or denture carried by the bridge with the gum, or the absence of pressure on the gum, is one of the advantages of the invention; while it was stated in the first application to be necessary 'to carefully fit the base of the tooth or block to be inserted to the jaw, and when secured, it should be so pressed down as to leave no space beneath it for the admission of food.' The statement in the first application is not inconsistent with the method of the patent, which consists in attaching the artificial tooth, or the denture, to bands and supporting them by the adjoining permanent teeth, 'without dependence upon the gum beneath'—so long as this essential feature of the invention is retained, it is quite immaterial whether the artificial dentition 'is so pressed down as to leave no space beneath it for the admission of food,' in the language of the specification, or whether it is in positive non-contact with the gum. When the artificial denture is in non-contact with the gum, cleanliness is facilitated, and the suggestion which was first made in the second application was, therefore, a useful one. But it did not change the invention in essentials. Although the tooth or denture is pressed down so close to the jaw that food cannot lodge between it and the gum, it is still supported by the adjoining tooth or teeth and not by the gum. As was stated in the first application, 'the yielding surface on which it rests will readily conform to the tooth or block, and any pain at first induced by the pressure will disappear.' There is nothing to indicate that Low intended to abandon his first application."

To further support the defence of anticipation, the defendant has introduced the same witnesses who, and the same exhibits which, were before the court in the Bennett case. The inexplicable contrast between the statements of the same persons in the two cases is either an object-lesson as to the fallibility of human memory, and the uncertainty of human testimony, or is forcibly suggestive of perjury and fraud. The only question discussed in the Richmond case was anticipation. Upon that point the opinion of the Circuit Court of Appeals contained, *inter alia,* the following statement:

"If the patent were valid the insertion of a single artificial tooth firmly secured to a band of gold accurately fitted and cemented to a natural tooth adjacent to the vacant space to be filled with such artificial tooth, and wholly supported by its attachment to such adjacent natural tooth without dependence on the gum beneath said artificial tooth, would be an infringement. If this were done before the application for the patent it would be an anticipation. The evidence that this is what was done in the case of Mrs. Martz is to our minds clear and convincing, the date is established beyond a doubt, and it is equally certain that the artificial tooth thus attached was used for years. We concur, therefore, with the judge who heard the cause in the Circuit Court that the so-called 'Beardslee-Martz 1877 Permanent Bridge' is an anticipation of the device of the patent."

The evidence in support of this finding is stated by Judge Wheeler, as follows:

"Dr. Beardslee testifies to making a similar cap of gold and attaching it to the natural tooth of a patient, wife of a clergyman, and to attaching at first an artificial tooth to one side of the cap, and afterwards another on the other side, which were worn, and gave satisfaction, several years. In this he is corroborated by an assistant, also learning the profession, and by the patient, her two daughters, and one of her Sunday-school scholars.

"There is nothing so improbable about this testimony, which is left wholly undisputed, as to leave any fair doubt as to the occurrences, or their date, both of which preceded Low's invention. The method of either seems to be the method of the patent and either seems to well have anticipated it."

In the present suit not one of these witnesses is able positively to identify said exhibit, and the "wife of a clergyman and her two daughters" now testify after an examination of church records, etc., that they were mistaken in their former testimony, and that the cap was not put into Mrs. Martz's mouth until 1878, or until after the Low invention was completed, as found in the Richmond case and further proved herein. Even Dr. Beardslee now says that he cannot now testify that said work was done any earlier than the year 1878, and that, so far as he knows, the testimony of the Martzes as to the date when it was done is correct. And further, as if to cap the climax of these contradictions, an apparently disinterested witness, Dr. Palmer, testified that he himself made the Beardslee-Martz exhibit, and was told at the time that "whatever of the kind I did was for use in defending the suit of the International Crown Company." It is unnecessary to further discuss this branch of the case.

At the conclusion of the first day's argument, counsel for complainant for the first time learned that the defendant herein was related to one of the officers of the complainant corporation, and that one of its stockholders had contributed to the defence herein without the knowledge of counsel for defendant. Counsel for complainant at once fully and frankly brought this matter to the attention of the court, and asked to be advised thereon. The questions thereby suggested have been borne in mind in consideration of the evidence herein. These circumstances, however, cannot relieve the court from its obligation to pass upon the question of fact presented by the evidence herein. The commendable frankness of counsel in the disclosure of these conditions has put the court on its guard against anything which might suggest collusion. The defence of anticipation herein is overwhelmingly disproved by disinterested witnesses. The methods by which the Beardslee-Martz evidence of anticipation was secured by Dr. Beardslee in the Richmond case appear to have been questionable and reckless, and it is hoped such practices are unusual. The contradictions in his own testimony are so direct and material as to disentitle him to any consideration. The Martz witnesses appear to have testified incorrectly as to the date when the work was done, because, as they say, "We were taken at such short notice we hadn't time to look accurately," or because "Dr. Beardslee was so sure that we came in 1877 that I thought it must be so," because they thought he had a record of such dates.

By reason of other contradictions in Beardslee's testimony, it appears that, even if the Beardslee-Martz device had been prior to the patent in suit, it would not have anticipated it, because it was supported on a root which was not taken out. Day's testimony has not been discussed because his veracity is attacked, his testimony is contradicted, and the facts stated by him, if true, would be insufficient for various reasons.

A decree may be entered for complainant for an accounting but not for an injunction, as the patent has expired.

DICKERSON & BROWN,
JAMES C. CHAPIN,
*For Complainant.*
ANDREW COMSTOCK,
*For Defendant.*

---

### KENTUCKY STATE DENTAL ASSOCIATION.

ATTENTION is called to the change of date of the meeting of the Kentucky State Dental Association. On account of change of meeting of Confederate Association, and for the purpose of getting railroad rates, we too have changed our date to May 29, 30, 31. We have some thirty papers promised for the meeting and nearly as many clinics, and we will still add others to the list.

F. I. GARDNER, D.D.S.,
*Secretary.*

LOUISVILLE, February 2, 1900.

---

### ILLINOIS STATE DENTAL SOCIETY.

THE thirty-sixth annual meeting will be held in Springfield, May 8 to 11, inclusive.

Drs. E. H. Allen, Executive Committee, and J. E. Hinkins, Supervisor of Clinics, promise an excellent programme, which will be printed in the May number of this journal.

All reputable dentists are cordially invited to attend.

A. H. PECK,
*Secretary.*

92 STATE STREET, CHICAGO.

### LEBANON VALLEY DENTAL SOCIETY.

The Lebanon Valley Dental Society will hold its annual meeting at the Allen House, Pottsville, Pa., May 15 and 16, 1900, to which all dentists are invited.

P. K. Filbert,
*Chairman Executive Committee.*

---

### MISSISSIPPI VALLEY MEDICAL ASSOCIATION.

The twenty-fifth annual meeting of the Association, at Chicago, was in every respect the most successful meeting in the history of the organization. Despite the many distractions of a large city, the meetings of the Sections were universally well attended, and a larger percentage of those down for papers were present to read them than ever before. The discussions were full and to the point, and on this account the volume of Transactions that will be issued at once will be very valuable. The following were appointed a Committee on Publication: Drs. Henry E. Tuley, Dudley S. Reynolds, and Lewis S. McMurtry. Those on the programme who have not handed in their papers must do so before November 1, after which time no paper will be received for publication.

The Executive Committee has ordered that no volume be sent a member who is in arrears for dues.

The following officers were elected for the coming year:

President, Dr. Harold N. Moyer, Chicago, Ill.; First Vice-President, Dr. A. H. Cordier, Kansas City, Mo.; Second Vice-President, Dr. S. P. Collings, Hot Springs, Ark.; Secretary, Dr. Henry E. Tuley, Louisville, Ky.; Treasurer, Dr. Dudley S. Reynolds, Louisville, Ky.; Chairman of Committee of Arrangements, Dr. M. H. Fletcher, Asheville, N. C.

Twenty-sixth annual meeting, Asheville, N. C., October 9, 10, 11, 1900.

Henry E. Tuley,
*Secretary.*

